# Role of imaging in morphea assessment: A review of the literature

**DOI:** 10.1111/srt.13410

**Published:** 2023-07-03

**Authors:** Faezeh Khorasanizadeh, Yasamin Kalantari, Ifa Etesami

**Affiliations:** ^1^ Department of Radiology Razi Hospital Tehran University of Medical Sciences Tehran Iran; ^2^ Department of Dermatology Razi Hospital Tehran University of Medical Sciences Tehran Iran

**Keywords:** elastography, localized scleroderma, morphea, MRI, sonography

## Abstract

**Background:**

Localized scleroderma, known as morphea, is a connective tissue disorder characterized by inflammation and fibrosis of the skin and the soft tissue. There exist no universally accepted validated outcome measures in order to monitor the disease activity. Besides clinical scores to evaluate outcome measures, imaging modalities are increasingly utilized in assessing patients with morphea, such as high‐frequency ultrasonography (US), shear‐wave elastography (SWE), and magnetic resonance imaging (MRI). However, the accuracy of these imaging modalities in monitoring morphea activity is not yet clear.

**Aims:**

To review the literature regarding the role of imaging modalities in assessing patients with morphea.

**Materials & Methods:**

In this study, we searched the PubMed/Medline database for articles published from inception until February 2023.

**Results:**

A total number of 23 original articles in three categories of US, elastography, and MRI were included.

**Discussion:**

Regarding US, criteria, including increased dermal thickness, increased echogenicity of the subcutaneous tissue, and decreased dermal echogenicity, were indicators of active morphea lesions when using high frequencies probe (18–20 MHz) color Doppler sonography. Moreover, studies evaluating SWE, a novel method to quantitatively assess tissue stiffness, demonstrated increased dermal stiffness in active lesions.

**Conclusion:**

Studies showed that MRI can help to determine the depth of disease, particularly as a first‐line and follow‐up diagnostic tool, especially in generalized and deep morphea. In addition, brain MRI may be useful for patients with localized craniofacial scleroderma experiencing new or worsening neurological symptoms.

## 1 INTRODUCTION

Localized scleroderma (LoS), also known as morphea, is a rare connective tissue disorder characterized by inflammation and fibrosis of the skin as well as the soft tissue that causes the imbalance of collagen production and destruction.^1^, ^2^, ^3^, ^4^ Although the exact pathogenesis of morphea remains unclear, it is believed that genetic predisposition, immune system dysregulation, trauma, and iatrogenic factors might be responsible for causing the disease.^1^, ^2^

Morphea, which is seen in all age ranges, is divided into four major subtypes: plaque type (circumscribed), linear, generalized, and mixed. Notably, plaque type and linear morphea are the most common types and main focus of most studies.^2^ The clinical presentation of morphea differs according to its subtype, degree of involvement, and stage of evolution. Active morphea lesions are characterized by erythema and induration and of note, most lesions of morphea have an insidious course and are asymptomatic. Inactive lesions show sclerosis and atrophy of the epidermis, dermis, and subcutaneous tissues.^2^

Due to the long‐lasting cutaneous and extra‐cutaneous manifestations of morphea, patients may experience serious aesthetic problems, tissue atrophy, functional disabilities, as well as muscular and neurological complications. Hence, early diagnosis of the active disease, staging, and response to the treatment is very important.^1^

Unfortunately, there exist no universally accepted validated outcome measures in order to monitor the disease activity.^4^ Disease activity in morphea is usually assessed by clinical evaluation, and biopsy is performed to diagnose the disease at base line. There exist some methods to evaluate outcome measures. Nevertheless, each of them has some limitations. For instance, some skin scores, such as the modified Rodnan skin score (mRSS), have not been validated for morphea. In addition, computerized surface area measurement is insensitive to improvement. Moreover, durometer has poor correlation with clinical skin scores, and its sensitivity to change is unknown. Cutometer has not been validated in morphea, and thermography is incapable of distinguishing between active and inactive phases.^4^

Some imaging modalities are utilized in assessing patients with morphea, such as high‐frequency ultrasonography (US), shear‐wave elastography (SWE), and magnetic resonance imaging (MRI). These imaging modalities are promising tools in the diagnosis and monitoring of morphea lesions. Furthermore, they seem to be more reliable and quantitative than other currently applied methods. However, there is no consensus on the definite monitoring criteria for morphea yet.^3^, ^4^

The aim of this study was to review the literature on the role of imaging modalities in assessing patients with morphea in terms of disease activity, discriminating between different phases, and response to the treatment.

## 2 METHODS AND MATERIALS

In this study, we searched the PubMed/Medline database for articles published from inception until February 2023 using the related keywords. Our search terms were “LoS,” “morphea,” “imaging,” “US,” “Elastography,” and “MRI.” Out of the 439 retrieved articles, 23 original studies were included in our study.

## 3 RESULTS

A total number of 23 original articles were enrolled in our study. In this study, we categorized our findings into three sections, and we reviewed the role of US, elastography, and MRI in morphea assessment. The characteristics of the included studies are described in Table 1.

**TABLE 1:** Characteristics of the included studies.

First author, year of publication, and reference number	Study type	Number of patients and lesions	Mean age (years)	sex	Study method	Equipment	Morphea lesions characteristics	Imaging outcome
**US**								
Jia, 2022,^32^	Comparative study	50 patients, 96 lesions	N/A	N/A	The skin thickness of the abdomen, chest, and left finger of the study and control groups was compared. The traditional NLM algorithm was improved by changing the Euclidean distance and introducing a cosine function, which was applied to the ultrasonic imaging intelligent diagnosis of patients with LoS. SWE imaging was evaluated	N/A	N/A	LS lesion edema stage accounted for 7.29%, hardening stage occupied 43.75%, and the proportion of atrophy stage reached 48.96%. When the size of shell was 1 mm, maximum elastic modulus (*E*_max_) was 0.984, mean of elastic modulus (*E*_mean_) was 0.926, and electro‐static discharge (*E*_sd_) was 0.965. When the size of shell was 2 mm, the elastic moduli around lesions were as follows: *E*_max_ was 0.998, *E*_mean_ was 0.968, and *E*_sd_ was 0.997. By comparing the skin thickness of the abdomen, chest, and left finger, it was found that there was a significant difference between the LS group and the control group (*p* < 0.05). When the shell was 2 mm, the effect of sensitivity specificity on SWE imaging was better than that when the shell was 1 mm
Marti‐Marti, 2022^31^	Cohort	31 morphea; 19 SS and 22 chronic cGVHD	52	Female (61.1%)	HFUS was conducted for patients. HFUS criteria for diagnosing active SD were increased Doppler vascularity and/or meeting all B‐mode gray scale ultrasonographic signs of activity. Discordance in SD activity between clinical and HFUS examination (D‐HFUS‐C) was evaluated at the time of the first ultrasonographic assessment. Changes in patients’ management were instituted after HFUS were recorded	An Esaote MyLab Class C (Esaote) US machine with 15, 18 and 22 MHz probes was used	In morphea, plaque subtype (61.3%)	In morphea cases, all ultrasonographic active lesions showed increased Doppler activity, whereas increased DT and decreased dermal echogenicity were observed in 42.9% (3/7) of cases, loss of definition of the dermal‐hypodermal junction in 57.1% (4/7) and hypoechogenicity and thickening of the septa or hyperechogenicity of the lobules in the hypodermis in 71.4% (5/7). Approximately half of the inactive lesions showed decreased DT (58.3%) and increased dermal echogenicity (41.7%) and almost none showed active HFUS findings
Laverde‐Saad, 2022^33^	Case report	1 patient	12	1 female (100%)	Sonography was performed for the patient at the base‐line, and two subsequent follow up after treatment	N/A	Linear type	Features suggestive of an active phase include a thickened and hypoechoic dermis contrasting hyperechoic subcutaneous tissue. The atrophic stage is characterized by a thinned‐out dermis and subcutaneous area. Typical vascular traits of each disease phase can also contribute to the assessment
Weibel, 2020^17^	Prospective	22 patients, 22 lesions	6.0 (range 0.2–14.4)	17 (77.27%) female, 5 (22.72%) male	Children with LoS were prospectively treated. Treatment response was evaluated by a clinical activity score. Skin temperature, blood flow, DT, and dermal echogenicity of clinically active skin lesions were determined in relation to the unaffected contralateral site at baseline and after 3, 6, 12, and 18 months	20 MHz US A‐mode scan (DermaScan, Cortex Technology)	Linear type: 21/22 (95.45%), deep: 1/22 (4.54%)	A weak positive correlation of dermal thickness and a weak negative correlation of echogenicity with clinical disease activity at treatment initiation were found. No obvious changes were detected in ultrasound parameters over time
Ranosz‐Janicka, 2018^10^	Prospective	40 patients, 92 lesions	49 ± 18 (range 34–65)	33 (82.5%) females, 7 (17.5%) males	Activity and damage in LoS lesions were assessed using LoSCAT and HFUS. Moreover, DT and echogenicity (intensity score, IS) of the LoS lesional dermis were measured and compared to the site‐matched unaffected skin	20 MHz US, A‐mode scan (DermaLab System Cortex Technology)	Atrophoderma idiopathica of Pierini and Pasini: 12/40 (30%), plaque: 11/40 (27.5%), generalized: 10/40 (25%), linear: 4/40 (10%), mixed: 3/40 (7.5%)	A high correlation between the clinical evaluation of the LoS lesions according to the LoSCAT and HFUS measurements was found. Clinical scores for erythema and skin thickness strongly had a positive relationship with the relative difference of DT at US. Furthermore, there was a significant relationship between increase in the DT and decrease in the echogenicity of the dermis with the inflammatory phase of the disease, increase in the DT as well as in the echogenicity of the dermis with the sclerotic phase, and decrease in the DT and increase in the echogenicity of the dermis with the atrophic phase
Arisi, 2019^13^	Prospective	17 patients, 17 lesions	64.05 (range 20–90)	16 (94.11%) female, 1 (5.88%) male	US was performed on the edge of a morphea lesion treated with UVA1 phototherapy before and after the study. An analysis of dermal features was performed and compared with the features of healthy skin. Skin biopsy samples were obtained from lesions at the beginning and end of the study, assessing dermal sclerosis and inflammatory infiltrate	50 MHz US A‐mode scan (DUB Skin Scanner, Taberna Pro Medicum)	GM: 14 (82.3%), DM: 3 (17.7%)	Compared to healthy skin, all affected skin showed a significant increase in DT and hypoechogenicity, undulations of the dermis in morphea lesions and in healthy skin areas were noted, whereas “yoyo” figures were more identified in morphea lesions compared to the healthy skin area. US morphological analysis showed improvement in dermal hyperechogenic bands and disappearance of yoyo figures after UVA1 treatment. Histology revealed a reduction in subcutaneous vascularity and dermal sclerosis and inflammation
Porta, 2014^16^	Prospective	10 patients, 10 lesions	101.7 ± 66.2 (range 35.5–167.9) months	3 (30%) female, 7 (70%) male	Patients were evaluated at baseline and after 6 months of treatment. The thickness of the lesions was scored at baseline and after 6 months with the mRSS and US dermis was analyzed at baseline and after 6 months; meanwhile, the hypodermis was only analyzed at baseline. Evaluation was also performed symmetrically in the contralateral healthy skin sites as controls	18 MHz US B‐mode scan (Esaote MyLab 70 XVG machine)	Circumscribed morphea: 3/10 (30%), linear scleroderma 3/10 (30%), and en coupe de sabre 4/10 (40%)	At baseline, there was a significant difference in mean DT measured with US at the center of the lesions compared to the normal skin in the contralateral area. With US, the hypodermis was significantly reduced in the lesional skin in respect to healthy skin. After treatment, in six out of the seven clinically improved patients, US detected a significant reduction of the thickening of the dermis. A positive correlation between the changes of mRSS and of the DT measured by US at sixth month was found
Wortsman, 2011^8^	Prospective	51 patients and 104 morphea lesions	28 (range 4–68)	37 (72.54%) female and 14 (27.45%) male	Lesions activity in US was compared with histologic findings	7–15 US B‐mode scan (HDI 5000 and IU 22, Philips)	Active lesions: 21/104 (20%); atrophic lesions: 23/104 (22%), and inactive lesions: 60/104 (58%)	The most definite sonographic signs of lesion activity were increased subcutaneous tissue echogenicity and increased cutaneous blood flow
Li, 2011^9^	Retrospective	21 patients and 52 lesions	12.2 ± 3.7 (range 4.6–18)	18 (85.71%) female and 3 (14.28%) male	Two physicians scored the scans using the U‐DA, which scores for differences in lesion echogenicity and vascularity compared with normal tissue. Tissue thickness differences were evaluated by percent differences and by using the TTS	8–14 MHz US B‐mode scan (ACUSON Sequoia 512 machine, Frequencies)	Linear type: 7/21 (33.33%), circumscribed: 6/21 (28.57%), generalized: 2/21 (9.52%), pansclerotic: 1/21 (4.76%) mixed: 5/21 (23.80%). Also, 13/21 (61.90%) were active and 8/21 (38.09%) were inactive	Most active skin lesions showed thinning of the dermis, whereas inactive skin lesions were more likely to show thickening or no difference. The majority of skin lesions, active and inactive, showed thinning of the hypodermis
Nezafati, 2011^11^	Prospective	14 patients, 16 lesions	32.07 (range 7–60) years	11 (78.57%) female, 3 (21.42%) male	Each patient and lesion was assessed for morphea subtype and clinical stage and was assigned a mRSS. A single site for US and biopsy, as well as a control site, was chosen	14 MHz US B‐mode	Atrophic: 3/16 (18.75%), inflammatory: 7/16 (43.75%), sclerotic: 6/16 (37.5%). Generalized: 4/16 (25%), lichen sclerosis/morphea generalized: 4/16 (25%), linear: 4/16 (25%), plaque: 4/16 (25%)	Inflammatory lesions were isoechogenic. Moreover, most sclerotic and atrophic lesions were hyperechogenic and hypoechogenic, respectively. No significant relationship between mRSS and US findings of DT or echogenicity was found. Dermal hyperechogenicity in US was significantly associated with the presence of moderate or extensive sclerosis on histopathology. There was not a significant relationship between the depth of sclerosis measured by US and histologic analysis
Cosnes, 2003^12^	Prospective	26 patients, 40 lesions	40 (range 6–67)	N/A	Morphea lesions were examined and compared blindly with 17 control plaques in 16 patients with skin diseases with differential diagnosis of LoS. Results were also compared with a normal control group. Five patients were reevaluated clinically and with ultrasound imaging 12–18 months after the first examination	13 MHz US B‐mode scan (ACUSON Sequoia 512 computed sonography system; ACUSON)	N/A	US revealed a characteristic dense image at the dermal‐hypodermal junction resembling a “flattened yo‐yo” with an acute angle at lateral limits similar to a “V.” This “yo‐yo” image is detected regardless of the duration of the plaque and can last for many years. There was a 92% sensitivity and a 100% specificity for LoS diagnosis when four of these five signs were present: Undulations of the dermis, disorganization, loss of thickness and thickened hyperechoic bands in the hypodermis, and the yo‐yo image
**Elastography**								
Pérez, 2020^20^	Prospective cross‐sectional	13 patients, 13 lesions	10 (range: 4 ± 17) years	10 (76.92%) female, 3 (23.07%) male	Conventional ultrasound and SWE imaging were used to characterize changes in pre‐assigned LS lesions. Contralateral sites were used as controls. The US protocol included three parts: gray scale B‐mode, color Doppler and SWE imaging	(ACUSON S2000, Siemens Medical Ultrasound Business Division)	linear 8/13 (61.53%), plaque 2/13 (15.38%), and mixed 3/13 (23.07%)	As compared to healthy skin sites before and after normalization of thickness, lesions with LoS exhibited an increase in stiffness in both the dermis and hypodermis
Wang, 2019^18^	Prospective	56 patients	43.48 ± 16.28 (13–72)	50/56 (89.29%) female, 6/56 (10.71%) male	DT, ARFI quality (elastography score) and quantity (mean SWV) were measured by US, diagnosis and stage performances of LS using the DT, elastography score and mean SWV compared with mLoSSI were evaluated. Moreover, 30 healthy controls were enrolled	6–18‐MHz US (EUB‐8500 US system Hitachi Medical) elastography with the S3000 US/(Siemens Medical Solutions)	Inflammatory 21/56 (37.5%), sclerotic 24/56 (42.85%), and atrophic 11/56 (19.64%). Limited: 18/56 (32.14%), generalized: 11 (19.64%), linear: 10 (17.86%), deep: 3 (5.36%), and mixed: 14 (25%)	The mean SWV of LS was significantly higher than that of normal subjects. Thicker derma in LS patients than in normal subjects without considering different skin sites was observed
Wang, 2017^19^	Prospective	21 patients, 37 lesions	19 (2–61)	12/21 (57.14%) female, 9/21 (42.85%) male	The pathologic stage (edema, sclerosis or atrophy) of the lesions was characterized by pathologic examination. The skin elastic modulus (*E*‐values including *E*_mean_, Emin, *E*_max_, and *E*_sd_) and thickness (*h*) was evaluated both in LS lesions and site‐matched unaffected skin (normal controls) using US‐SWE. The relative difference of *E*‐values (ERD) was calculated between each pair of lesions and its normal control for comparison among different pathologic stages	4–15 MHz US (Aixplorer, SuperSonic Imagine)	Limited type: 12/21 (57.14%), generalized type: 2/21 (9.52%), linear type: 6/21 (28.57%), and deep type: 5/21 (23.80%)	A significant increase in skin stiffness was measured by US‐SWE in LS lesions compared with normal skin controls
**MRI**								
Abbas et al.^25^	Cross‐sectional	20 patients	N/A	15/20 (75%) female, 5/20 (15%) male	Clinical assessment was done using Localized Scleroderma Assessment Tool (including modified mLoSSI, PGA‐A, LoSDI, and Physician Global Assessment of Disease Damage). The results of the clinical evaluation were compared to the MRI findings	MRI device	Linear morphea: 14 (70%)	MRI results revealed clinically occult musculoskeletal involvement. Moreover, the validated clinical parameters used to determine activity as part of the mLoSSI score did not correspond to features of activity on MRI findings, and in 5 patients (19%) MRI results demonstrated occult active disease
Maloney, 2018^28^	Retrospective study	10 patients	6.2 (range 1–16) years	8/10 (80%) female, 2/10 (20%) male	A search of radiology reports was performed using related keywords. CT, MRI, and PET brain examinations were selectively identified, and reports were reviewed for any described intracranial abnormalities	MRI, CT, and PET devices	Linear morphea: 9/10 (90%)	Hyperintense white matter lesions ipsilateral to the cutaneous lesion was the most common neuroimaging abnormality. Abnormal T2‐hyperintense foci were the most commonly observed neuroimaging abnormality. Calcifications or blood products were noted in five cases, cysts in four and abnormal enhancement in two. Progressive neuroimaging abnormalities were documented in three cases
Shahidi‐Dadras, 2018^26^	Trial	33 patients	29.00 (10–61) years	28/33 (84.84%) female and 5/33 (15.15%) men	Patients received 15 mg/week of MTX and monthly pulses of methylprednisolone for 3 days in 6 months. The effectiveness of the treatment was evaluated by MRI, mLoSSI, and LoSDI	MRI device	Linear morphea: 28/33 (84.84%), generalized 5/33 (15.15%)	Subcutaneous fat enhancement was the most common finding in MRI. MRI scores were significantly associated with clinical markers both before and after the treatment with the exception of skin thickness and new lesion/lesion extension which were not associated with MRI scores before and after the treatment, respectively
Eutsler, 2017^23^	Retrospective study	14 patients	9 (range 2–17) years	4/14 (28.57%) female, 10/14 (71.42%) male	20 MRI studies of the extremities in 14 children with juvenile LoS were evaluated	MRI device	Linear scleroderma: 6/14 (42.85%), GM: 4/14 (28.57), mixed morphea: 3/14 (21.42%), circumscribed morphea: 1/14 (7.14%)	Deep tissue involvement was detected in 65% of the imaged extremities. Fascial thickening and enhancement were seen in 50% of imaged extremities. Axial T1, axial T1 FS contrast‐enhanced, and axial fluid‐sensitive sequences were rated most useful
Doolittle, 2015^30^	Retrospective study	88 patients	28.8 years	62/88 (70%) female, 26/88 (29.54%)	Patients younger than 50 years at our institution over a 16‐year interval who had clinical diagnosis of Parry–Romberg syndrome and ECDC by a skin or facial subspecialist were reviewed. Two neuroradiologists evaluated available imaging and characterized CNS imaging findings	CT, MRI, CTA, MRA, and conventional angiography	N/A	The most common CNS imaging finding was white matter T2 hyperintensity, and areas of T2 hyperintensity were subcortical in these patients. Moreover, association with atrophy or encephalomalacia were observed
Schanz, 2013^24^	Prospective study	22 patients	52 years	16/22 (72.72%) female, 6/22 (27.27%) male	Patients with systemic scleroderma and DM prospectively underwent whole‐body MRI twice, before treatment (time 1) and during follow‐up after 6–12 months (time 2). Images were evaluated on STIR and gadolinium‐enhanced scans. Moreover, the LoS (morphea) severity index and a 0–6 pain score were applied	1.5‐T closed‐bore whole‐body MRI scanner (Avanto, Siemens Healthcare) equipped with parallel‐imaging technology of up to 32 independent receiver channels (total imaging matrix technique, Siemens Healthcare)	Circumscribed DM 3/22 (13.63%), linear morphea 7/22 (31.81%), GM 11/22 (50%), pansclerotic morphea 1/22 (4.54%)	MRI findings were sensitive to changes in musculoskeletal manifestations in patients with DM undergoing systemic treatment
Chiu, 2012^29^	Retrospective study and literature review	32 patients	9.3 (3–17)	21/32 (66%) female, 11/32 (34%) male	Cases of ECDS and Parry–Romberg syndrome in an institution were reviewed. Moreover, 51 additional cases from the literature were reviewed	MRI, CT	Linear morphea: 32/32 (100%)	Hyperintensities on T2‐weighted sequences were the most common finding, present in all children with intracranial abnormalities on MRI
Schanz, 2011^22^	Prospective	43 patients	42 (range)	30/43 (69.76%) female, 13/43 (30.23%) male	Patients with LS underwent MRI. Findings were classified into clinical subtypes. Images were evaluated for morphologic changes and signal abnormalities. Clinically suspicious features of musculoskeletal manifestations were recorded	1.5‐T closed‐bore whole‐body MR imager (Avanto; Siemens) equipped with parallel imaging technology and up to 32 independent receiver channels (total imaging matrix technique)	Circumscribed DM: 9/43 (20.93%), linear scleroderma 19/43 (44.18%), GM 12/43 (27.90%), pansclerotic morphea: 3/43 (6.97%)	Musculoskeletal involvement was detected in a majority of patients. Fascial thickening, increased fascial enhancement, articular synovitis, tenosynovitis, perifascial enhancement, myositis, enthesitis, bone marrow involvement, and subcutaneous septal thickening. The highest prevalence of musculoskeletal involvement was seen in patients with pansclerotic morphea
Liu, 1994^27^	Retrospective	23 patients	11.1 (2–17)	13/23 (56.52%) female, 10/23 (43.47%) male	The imaging studies of patients with the clinical diagnosis of LS were reviewed	CT, MRI, plain radiographs, upper gastrointestinal and small bowel follow‐through studies	N/A	The most common findings were leg length discrepancy and muscle atrophy

Abbreviations: ARFI, acoustic radiation force impulse; cGVHD, graft‐versus‐host disease; CT, computed tomography; CTA, computed tomographic angiography; DM, deep morphea; DT, dermal thickness; ECDC, en coup de sabre; FS, fat suppressed; GM, generalized plaque morphea; HFUS, high‐frequency ultrasound; LoS, localized scleroderma; LoSCAT, Localized Scleroderma Cutaneous Assessment Tool; LoSDI, localized scleroderma damage index; mLoSSI, modified localized scleroderma skin severity index; MRA, magnetic resonance angiography; MRI, magnetic resonance imaging; mRSS, modified Rodnan skin score; NLM, nonlocal means; PET, positron emission tomography; PGA‐A, physician global assessment of disease activity; SS, systemic sclerosis; SWE, shear‐wave elastography; SWV, shear‐wave velocity; TTS, tissue thickness score; U‐DA, ultrasound disease activity; US, ultrasonography; UVA1, ultraviolet A‐rays.

### 3.1 Ultrasonography

Ultrasound imaging is a noninvasive and objective method for evaluating morphea. The combination of US and Doppler imaging allows for the evaluation of both superficial and deep soft tissue thicknesses, echogenicity as well as subcutaneous vascularity of skin lesions in LoS. By using high‐frequency ultrasound, quantitative assessments with higher resolutions can be performed.^5^, ^6^, ^7^ The study done by Serup et al. was the first ultrasonographic evaluation of morphea.^8^

Color Doppler ultrasound can accurately assess morphea activity. Wortsman et al. evaluated 104 morphea lesions using a compact linear probe 7–15 MHz and established that morphea activity can be assessed by using sonographic criteria. Active lesions need two or more of the first three following criteria: increased dermal thickness (DT), increased echogenicity of the subcutaneous tissue, and decreased dermal echogenicity. Notably, increased cutaneous blood flow is a marker of morphea activity itself. Incomplete criteria for active lesions indicate an inactive lesion. Moreover, atrophic lesions are characterized by decreased subcutaneous and DT, without an increase in the blood flow. In comparison with histology as the gold standard, ultrasound was 100% sensitive and 98.8% specific in its ability to differentiate between activity phases. Increased subcutaneous tissue echogenicity and increased cutaneous blood flow are the most accurate indicators of the lesion activity (sensitivity and specificity 100%). Moreover, all five patients with Parry–Romberg syndrome in the study showed signs of ipsilateral parotid gland inflammation as diffuse hypoechogenicity, small hypoechoic nodules, and increased blood flow.^9^

Li et al. evaluated the construct validity of two proposed measures; the ultrasound disease activity and the tissue thickness score by using 8–14 MHz probe on 52 lesions of 21 juvenile LoS cases and concluded that there were significant differences in the echogenicity and vascularity of the hypodermis and deep tissues between the active and inactive lesions. Moreover, there were no significant differences between the dermis and other layers in terms of echogenicity or vascularity. However, the researchers suggested that monitoring thickness changes may help to identify the response to treatment because lesions that show persistent thickening or thinning are more likely to be active than those with a static thickness.^10^

Twenty MHz US assessment of DT and echogenicity in 92 morphea lesions indicated that there is a significant correlation between the inflammatory phase of the disease and an increase in the DT as well as a decrease in the dermis echogenicity. Moreover, there is a correlation between the sclerotic phase of the disease and an increase in the DT as well as an increase in the dermal echogenicity, and between the atrophic phase and a decrease in the DT as well as an increase in the dermal echogenicity. In comparison with the LoS Cutaneous Assessment Tool (LoSCAT) criteria for active and inactive diagnoses, ultrasound was 96.77% sensitive and 90.16% specific. The study did not evaluate hypo DT and echogenicity.^11^

In a study of 16 lesions using 14 MHz US, inflammatory lesions were isoechogenic. Moreover, most sclerotic and atrophic lesions were hyperechogenic and hypoechogenic, respectively. No significant relationship between mRSS and US findings of DT or echogenicity was found. Dermal hyperechogenicity in US was significantly associated with the presence of moderate or extensive sclerosis on histopathology. There was not a significant relationship between the depth of sclerosis measured by US and histologic analysis. The study did not evaluate hypoderm thickness and echogenicity as well.^12^

Using a 13 MHz probe, the morphological evaluation of 40 morphea lesions revealed a characteristic dense image at the dermal‐hypodermal junction resembling a “flattened yo‐yo” with an acute angle at lateral limits similar to a “V.” This “yo‐yo” image is detected regardless of the duration of the plaque and can last for many years. There was a 92% sensitivity and a 100% specificity for LoS diagnosis when four of these five signs were present: undulations of the dermis, disorganization, loss of thickness and thickened hyperechoic bands in the hypodermis, and the yo‐yo image.^13^

According to the Arisi et al.’s study using 50 MHz ultrasound on 17 lesions, there is a reduction in dermal density as well as an increase in DT in morphea‐affected sites. The morphological evaluation identified undulations of the dermis and “yoyo” figures. The study did not evaluate hypo DT and echogenicity, too.^14^

Additionally, US can be used to evaluate clinical improvement after the treatment. In two previous studies, 20 MHz ultrasound examinations showed a decrease in DT and an increase in the dermal density after treatment.^15^, ^16^ In this regard, Porta et al. monitored DT of 10 morphea lesions at baseline and after 6 months of treatment using 18 MHz US and concluded that a reduction in the DT can be suggestive of the disease improvement.^17^ In contrast, 50 MHz US quantitative analysis did not show any significant improvement in DT or density after ultraviolet A1 phototherapy, whereas morphological analysis highlighted an improvement in dermal hyperechogenic bundles and disappearance of yoyo figures meaning that in terms of therapeutic response evaluation, pattern analysis is more useful than quantitative analysis.^14^ Similar to Arisi et al. study, Weibel et al. followed 22 children with LoS using a 20 MHz probe and did not observe any obvious changes in ultrasound parameters over time. However, there was a weak positive correlation between DT as well as a weak negative correlation between echogenicity and clinical disease activity at the time of treatment initiation. They suggested that despite the clinical improvement, ultrasound structural changes of previously affected areas can last for long periods, or even forever.^18^

There are discrepancies in available literature because of different cross‐sectional study designs, various probe frequencies, and small sample sizes, lack of sufficient follow‐up study by US and using A‐mode (amplitude mode) scan in some studies which give one dimension plotted images in compared to 2D images in the B‐mode (brightness mode) scan which have higher resolution and simple to interpreted (Table 1).

**FIGURE 1:**
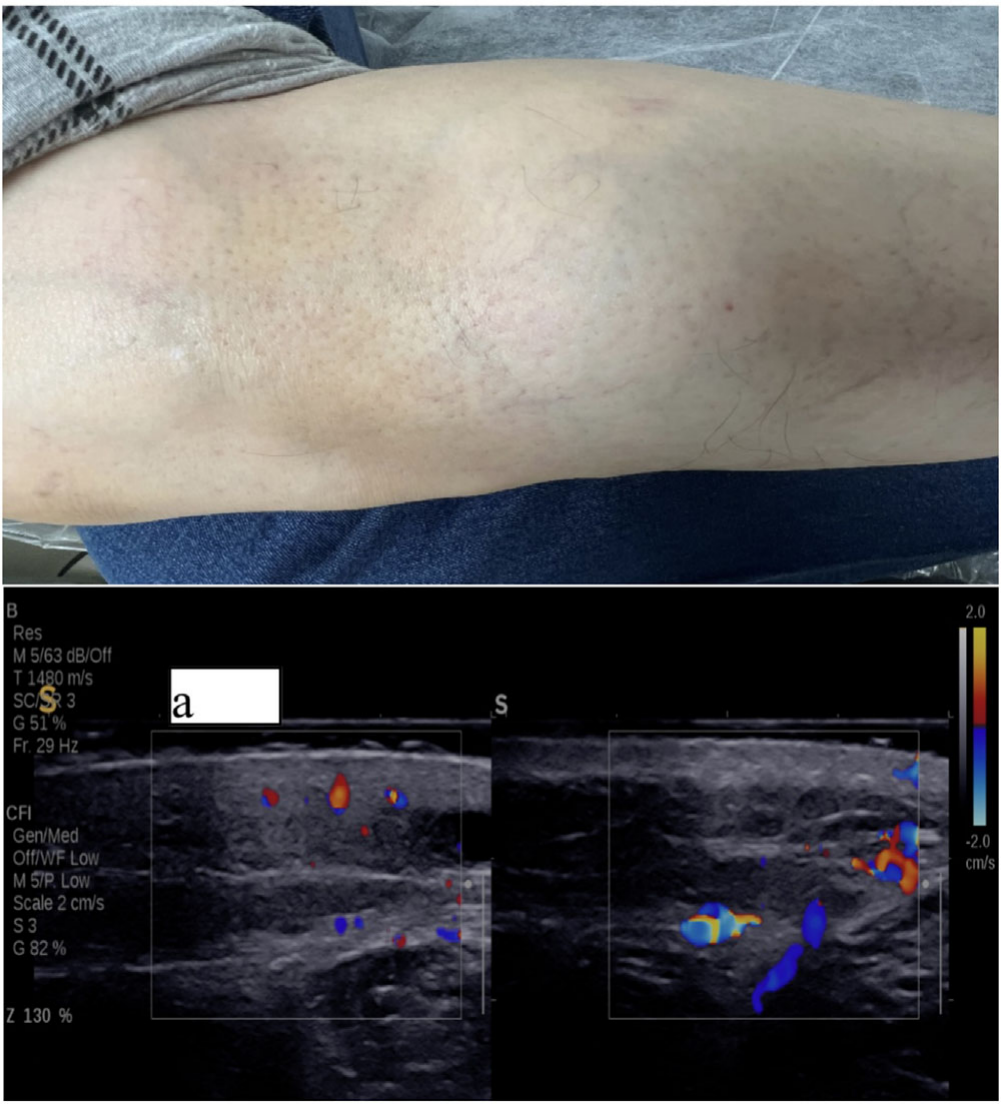
Color Doppler ultrasound images of morphea plaque in infrapatellar subcutaneous region of 50‐year‐old male showing dermal thickening and hypoechogenicity, as well as increased echogenicity of hypoderm causing dermal‐hypodermal blurredness and increased subcutaneous vascularity comparing adjacent normal soft tissue in favor of active plaque.

In our institution as a referral dermatologic center, we use both gray scale and color Doppler B‐mode ultrasound performed with Aixplorer (SuperSonic Imagine) using 20 MHz probes after diagnosis confirmation of morphea by biopsy and then follow the patients in 3, 6 months, and 1 year interval and report baseline dermal and hypo DT and echogenicity and subcutaneous vascularity in suspicious area of activity based on dermatologist judgment and compare it with normal adjacent or contralateral soft tissue then give a final comments as active, inactive or atrophic plaque based on Wortsman et al.^8^ criteria mentioned above, and then dermal and hypo DT and echogenicity of plaques as well as subcutaneous vascularity measure in follow‐up exam for the evaluation of disease progression or regression (Figure 1).

The most challenging part of ultrasound for follow up is that dermal and especially hypo DT are variable in even one plaque and track thickness difference need to follow the exact location of previous site of US, necessitate the use of picture archiving system for defining the plaque for follow up. Another potential limitation is that sometimes it is impossible to define a similar normal soft tissue at ipsilateral or contralateral side to compare in patients with extensive disease.

### 3.2 Elastography

Elastography is a novel method to quantitatively assess tissue stiffness and is performed in two ways: strain elastography (SE) and SWE. SE measures the amount of deformation and could be operator‐dependent and rather qualitative. In contrast, SWE uses acoustic radiation force impulses to generate shear waves and measures the speed of wave propagation to directly calculate the tissue's stiffness expressed in kilopascals (kPa). It gives quantitative information about tissue elasticity properties and is operator‐independent. Recently, the SWE method has been used for the assessment of skin‐involved diseases, such as systemic sclerosis and morphea.^19^

In a study done by Wang et al., shear‐wave elastography was compared with the clinical score of patients with LoS. Fifty‐six patients with LoS and 30 healthy patients were evaluated by using an 18‐MHz US probe frequency. The results showed that a thicker dermis, a higher elastography score, and a higher mean shear‐wave velocity (SWV) might be suggestive of LoS. Regardless of the lesion site, the mean SWV was more than 2.98 m/s for LoS and was less than 3.78 m/s for the inflammatory stage, more than 3.78 m/s for the sclerotic stage, and more than 6.01 m/s for the atrophic stage. Comparing the area under the receiver–operator curves of DT, elastography score, SWV, and modified localized scleroderma skin severity index (mLoSSI), SWV was the most useful tool for diagnosing and staging LoS.^19^

Study on 21 patients with LoS shows increased skin stiffness in shear‐wave elastography. Two of 21 patients had edematous lesions, and skin thickness was 193% higher in these two patients. Moreover, before and after skin‐thickness normalization, the skin stiffness was higher and lower than that of normal patients, respectively. Due to the low sample size, the skin elastic modulus of edematous lesions was excluded for further investigations. On average, the sclerotic lesions were 40% thicker than normal controls, whereas the atrophic lesions were 30% thinner than healthy skin. Elastic‐values (*E*_mean_, *E*_min_, and *E*_max_) were significantly greater in LoS lesions both for sclerosis and atrophy compared to the healthy skin before or after skin‐thickness normalization. Sclerotic lesions exhibited a significantly higher stiffness compared to atrophic lesions. After skin thickness‐normalization, opposite results were obtained and atrophic lesions exhibited significantly greater stiffness than sclerotic lesions. Accordingly, as the disease progression affects both elasticity and skin thickness, the normalized skin modulus may represent the true stiffness of the affected skin more accurately. This may account for the lower skin stiffness in the two patients with edematous lesions as the skin was thicker in the edematous stage.^20^

In this regard, Pérez et al. evaluated 13 patients with pediatric‐onset LoS, suggesting that SWE is a useful technique for determining affected skin sites. As compared to healthy skin sites before and after normalization of thickness, lesions with LoS exhibited an increase in stiffness in both the dermis and hypodermis. Due to the low sample size, they did not analyze different stages of the disease and also did not explore the association between skin stiffness and pathologic findings.^21^

**FIGURE 2:**
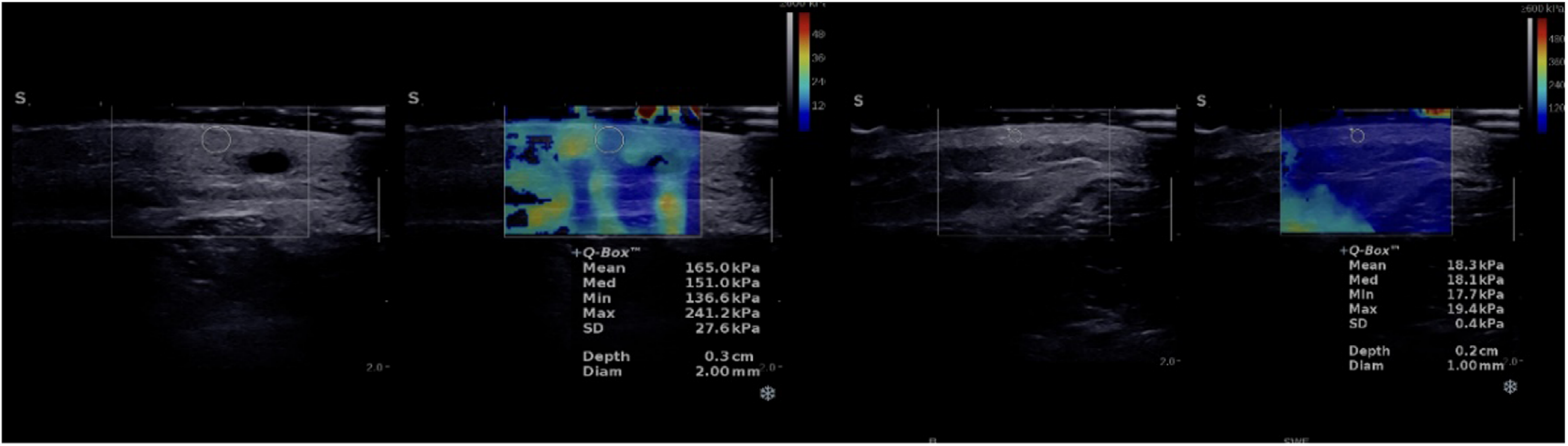
soft tissue shear‐wave elastography shows increased dermal elasticity compared to adjacent normal soft tissue.

All of the abovementioned studies are cross‐sectional and did not follow skin stiffness by SWE ultrasound after treatment. We conducted a cohort study of morphea patients and experimentally use shear‐wave elastography performed with Aixplorer (SuperSonic Imagine) using 20 MHz probe and calculate dermal and hypodermal elasticity by using copious amount of gel and minimal hand pressure and report tissue stiffness average of three measurements express in kPa for normal and abnormal dermis and hypodermis at baseline and then follow them in 3 and 6 months interval after treatment (Figure 2). Data will be published soon.

### 3.3 Magnetic resonance imaging (MRI)

MRI has been shown to be a valuable diagnostic tool for detecting and monitoring musculoskeletal involvement in morphea patients given its excellent soft‐tissue contrast and lack of ionizing radiation. The study done by Zemtsov et al. was a seminal paper about the use of MRI in dermatology.^22^

Imaging features of morphea are not specific and overlap with other disorders that involve skin, fascia, and muscle, such as some types of fasciitis and myositis. Nevertheless, MRI enables a complete assessment of the disease, including the depth of infiltration and the disease activity.^23^ Zemtsov et al. confirmed an excellent correlation between MRI and histology.^24^

The results of a previous study on MRI findings of 43 patients with LoS excluded the superficial subtype which is confined to the epidermis and dermis detected musculoskeletal involvement in 74% of patients. MRI detected musculoskeletal involvements in 96% of the clinically suspected patients and in 38% of not clinically suspected patients. They found “fascial thickening” as the most common musculoskeletal MRI finding (60%). Moreover, increased fascial enhancement, articular synovitis, tenosynovitis, and subcutaneous septal thickening were the most prevalent findings. Moreover, they found that all pansclerotic morphea patients had articular synovitis.^25^

In a recent study, MRIs of the extremities in 14 children with juvenile localized scleroderma were evaluated. Deep tissue involvement was detected in 65% of them. Fascial thickening and fascial enhancement were the most common findings of deep tissue involvement. Besides, infiltration and atrophy of the subcutaneous fat were the most prevalent subcutaneous finding. Moreover, muscle and bone marrow edema were seen in 40% and 20% of the images, respectively. In six patients, subsequent imaging after treatment was done in 2–5 years of follow‐up, showed improvement but not necessarily resolution of the superficial and deep involvement. They concluded that axial T1, axial fluid‐sensitive, and axial T1‐FS contrast‐enhanced sequences should be included in the imaging protocol, and the findings that were detected in contrast‐enhanced sequences were also seen in fluid‐sensitive sequences, meaning that intravenous contrast is not necessary for these examinations.^26^

In this regard, in a study performed by Schanz et al., 22 patients with systematic scleroderma and deep morphea (DM) underwent whole‐body MRI twice, once before treatment and once after receiving methotrexate and prednisone for 6–12 months. MRI scores for subcutaneous and fascial thickening as well as enhancement were significantly lower among those who responded to the treatment. The researchers concluded that MRI can detect changes in musculoskeletal manifestations during the treatment. Thus, MRI can be recommended as a complementary diagnostic tool for monitoring and assessing the clinical response of patients with morphea.^27^

Abbas et al. concluded that when using clinical examination solely, lesion's activity might be underestimated. In this regard, the results of MRI demonstrated occult active disease in 19% of patients could change the treatment regimen resulting in continuation or escalation. These discrepancies suggest that by using the mLoSSI alone, active lesions might be underestimated as inactive ones, especially in DM where the activity is difficult to detect. In comparison with the MRI, palpable peripheral induration that is a component of the mLoSSI can properly reflect inflammation. This finding suggests that induration is a sign of activity especially in deeper tissues where erythema is not visible. MRI imaging also revealed the subclinical extension of lesions, indicating that the pathology extends beyond clinical margins.^28^

In Shahidi et al. study, 33 patients with morphea were given methotrexate and pulses of methylprednisolone. They mentioned that cutaneous fat enhancement was the most prevalent finding in MRI. Then, the effectiveness of the treatment was examined by MRI. They concluded that the clinical markers of mLoSSI and LoSDI except for skin thickness and new lesion extension were in association with MRI scores before and after the therapy. However, they did not explain other MRI findings or MRI scoring in detail.^29^

Liu et al. evaluated 23 patients with morphea mainly linear type and revealed positive findings in half of the patients. They reported leg length discrepancy, muscle atrophy, bone remodeling deformity, abnormal bone marrow signals, and joint contracture as the main peripheral findings. They also reported intracranial calcifications and white matter abnormalities. They also found central nervous system abnormalities in 3 patients with “en coup de sabre” deformity that were neurologically asymptomatic.^30^

Furthermore, neuroimaging manifestations of localized craniofacial scleroderma are reported by several authors. T2‐hyperintense subcortical white matter lesions ipsilateral to the cutaneous lesion are the most common finding in localized craniofacial scleroderma. Gray matter involvement, calcifications, cysts, ventricular asymmetry, and abnormal parenchymal or leptomeningeal enhancement are other findings reported in localized craniofacial scleroderma. Neuroimaging abnormalities were reported in the absence of neurological symptoms. No consistent relationship between changes in neurological symptoms after the treatment and neuroimaging findings has been detected.^31^, ^32^, ^33^

## 4 CONCLUSION

One of the most challenging issues in treating morphea patients is the lack of globally accepted assessment tools for monitoring the results of treatment and disease activity.^33^, ^34^, ^35^, ^36^ In this regard, ultrasound is a potential and reliable imaging modality in the quantitative morphea assessment. However, ultrasound has limitations such as being operator dependent and lack of consistent intra or inter‐operator measurement of tissue thickness. It should be considered that there are discrepancies in available literature because of different cross‐sectional study designs, using A‐mode scan and various probe frequencies, and small sample sizes. To establish a quantitative, valid, and reproducible outcome measure, larger prospective cohort studies using higher probe frequencies should be conducted. In addition, shear‐wave elastography can be used for disease monitoring and assessing therapeutic effects. SWE is a novel tool that is not universally available and it is not FDA approved for skin so far. To determine the role of SWE in detecting lesion progression and changes overtime, future studies are needed.

MRI provides complementary information about the depth of the disease especially in deep or generalized morphea (GM) as well as neurologic manifestations of localized craniofacial scleroderma. MRI has also limitations, including being expensive, time‐consuming, infeasible in routine clinical settings, and a low signal‐to‐noise ratio for superficial layers intrinsically.

In conclusion, we recommend color Doppler ultrasound with high frequencies probe (18–20 MHz) and if available, SWE for assessing and monitoring superficial soft tissue involvement. In GM or DM, MRI helps to determine the depth of disease, particularly as a first‐line and follow‐up diagnostic tool. In addition, brain MRI may be useful for patients with localized craniofacial scleroderma experiencing new or worsening neurological symptoms.

## CONFLICT OF INTEREST STATEMENT

The authors declare that there is no conflicts of interest that could be perceived as prejudicing the impartiality of the research reported.

## Data Availability

Research data are not shared.
